# Physician Perspectives on Internet-Informed Patients: Systematic Review

**DOI:** 10.2196/47620

**Published:** 2024-06-06

**Authors:** Qianfeng Lu, Peter Johannes Schulz

**Affiliations:** 1 Faculty of Communication, Culture and Society Università della Svizzera italiana Lugano Switzerland; 2 Department of Communication & Media Ewha Womans University Seoul Republic of Korea; 3 Wee Kim Wee School of Communication and Information Nanyang Technological University Singapore Singapore

**Keywords:** internet-informed patients, physician-patient communication, health information–seeking, misinformation, digital health

## Abstract

**Background:**

The internet has become a prevalent source of health information for patients. However, its accuracy and relevance are often questionable. While patients seek physicians’ expertise in interpreting internet health information, physicians’ perspectives on patients’ information-seeking behavior are less explored.

**Objective:**

This review aims to understand physicians’ perceptions of patients’ internet health information-seeking behavior as well as their communication strategies and the challenges and needs they face with internet-informed patients.

**Methods:**

An initial search in PubMed, Scopus, CINAHL, Communication and Mass Media Complete, and PsycINFO was conducted to collect studies published from January 1990 to August 1, 2022. A subsequent search on December 24, 2023, targeted recent studies published after the initial search cutoff date. Two reviewers independently performed title, abstract, and full-text screening, adhering to PRISMA (Preferred Reporting Items for Systematic Reviews and Meta-Analyses) statement guidelines. Thematic analysis was then used to identify key themes and systematically categorize evidence from both qualitative and quantitative studies under these themes.

**Results:**

A total of 22 qualifying articles were identified after the search and screening process. Physicians were found to hold diverse views on patients’ internet searches, which can be viewed as a continuous spectrum of opinions ranging from positive to negative. While some physicians leaned distinctly toward either positive or negative perspectives, a significant number expressed more balanced views. These physicians recognized both the benefits, such as increased patient health knowledge and informed decision-making, and the potential harms, including misinformation and the triggering of negative emotions, such as patient anxiety or confusion, associated with patients’ internet health information seeking. Two communicative strategies were identified: the participative and defensive approaches. While the former seeks to guide internet-informed patients to use internet information with physicians’ expertise, the latter aims to discourage patients from using the internet to seek health information. Physicians’ perceptions were linked to their strategies: those holding positive views tended to adopt a participative approach, while those with negative views favored a defensive strategy. Some physicians claimed to shift between the 2 approaches depending on their interaction with a certain patient. We also identified several challenges and needs of physicians in dealing with internet-informed patients, including the time pressure to address internet-informed patient demands, a lack of structured training, and being uninformed about trustworthy internet sites that can be recommended to internet-informed patients.

**Conclusions:**

This review highlights the diverse perceptions that physicians hold toward internet-informed patients, as well as the interplay between their perceptions, communication strategies, and their interactions with individual patients. Incorporating elements into the medical teaching curriculum that introduce physicians to reliable internet health resources for patient guidance, coupled with providing updates on technological advancements, could be instrumental in equipping physicians to more effectively manage internet-informed patients.

**Trial Registration:**

PROSPERO CRD42022356317; https://www.crd.york.ac.uk/prospero/display_record.php?RecordID=356317

## Introduction

### Background

The internet has become a common resource for people seeking health-related information [1,2]. It offers a vast array of health information that was previously accessible mainly through physicians. Over the past decade, attention to internet health information–seeking behavior among patients has grown. The idea of patients searching for information on the internet can be traced back to Ferguson [3], who coined the term *e-patients* in 2007 for those seeking health information on the internet.

People seeking health information on the internet may have different purposes [4]. Before seeing a physician, they might search on the internet to determine if a medical appointment is necessary. Once a consultation is scheduled, some patients look for background information to better prepare for their appointments [5]. After consultations, some turn to the internet to clarify and supplement the physician’s information, such as details about medication indications and side effects [6]. These patients seek internet health information to better understand their diagnosed condition and manage their treatment [7]. Being equipped with knowledge from the internet, they are often referred to as internet-informed patients [8].

Some patients intentionally avoid information from the internet. For instance, information avoidance among cancer patients has been frequently explored by researchers [9,10]. However, several systematic reviews from patients’ perspectives conclude that patients often feel more confident discussing their health with physicians and feel more empowered to manage their conditions after searching for information on the internet [11-13]. Internet health information allows patients to transition from being passive and uninformed recipients to empowered and informed consumers [14,15].

However, searching for health information on the internet poses challenges for both patients and the health care system. The vast amount of internet health information can be overwhelming for laypeople, who might find it difficult to sift through and identify relevant and accurate content. Consequently, patients may end up learning mis- and disinformation or become confused by conflicting information they encounter on the internet [16,17]. Seeking internet health information can exacerbate patients’ anxiety about their health [18,19]. In contrast, physicians can provide accurate and personalized information, which can reassure patients. This becomes particularly vital in cases where patients, having been misinformed by internet sources, are taking active steps to manage their health [20].

Although the internet has become a popular source for accessing health information, consultations with physicians remain the primary influence on patients’ medical decisions [21]. Patients have various strategies for addressing the internet health information they gather before a consultation. They might either implicitly or explicitly share this information, hoping the physician will consider it in their judgment [22]. However, some may withhold it, fearing it might upset their physician [13]. A significant barrier to such disclosure is the concern that the physician might perceive it as a challenge to their expertise [23]. Many patients are cautious about revealing internet information to their physicians to avoid causing offense [24]. As a result, they often assess the physician’s potential reactions to such information before deciding whether to share it [25]. Research has shown that dialogue about internet information between patients and physicians can improve patient satisfaction and their relationships. This improvement is particularly noticeable when physicians acknowledge the patients’ efforts and take their internet-sourced information seriously [26].

The physician-patient relationship is dyadic, meaning it is built on mutual trust and efficient communication at its core [27]. Patients often access health information through the internet and approach physicians with a desire to seek clarification and advice [28]. Discussing internet health information offers a new communication opportunity, allowing for physicians to better understand their patients’ preferences and concerns and for patients to deepen their trust in their physicians. To maximize this communication opportunity, understanding the perspectives of both parties, that is, physicians and internet-informed patients, is crucial. However, while much of the literature focuses on internet-informed patients, there is less emphasis on physicians’ perspectives [29]. Several reviews have explored patients’ internet health information–seeking behavior and its impact on the physician-patient relationship [11,13,14,30,31]. Nonetheless, these reviews primarily focused on patients, with evidence collected mainly from the patient’s viewpoint.

### Objective

To the best of our knowledge, this is the first systematic review focusing on physicians’ perspectives. We aimed to explore physicians’ views on patients’ internet information–seeking behavior and their experiences with internet-informed patients. Our research questions (RQs) are elaborated as follows:

- RQ1: how do physicians perceive patients’ internet health information–seeking behavior?
- RQ2: what communicative strategies do physicians use in interacting with internet-informed patients? In this context, the term *strategies* refers to physicians’ actions and responses to internet-informed patients.
- RQ3: what difficulties and needs do physicians encounter when dealing with internet-informed patients?

## Methods

### Search Strategy

This systematic review was conducted in accordance with the PRISMA (Preferred Reporting Items for Systematic Reviews and Meta-Analyses) statement [32,33].

To identify the appropriate search algorithm, we undertook 3 steps. In the first step, we conducted an exploratory search using various combinations of terms such as *physician*, *communication strategies*, *internet-informed patients*, and *physician-patient communication*. Upon reviewing the reference lists of several primary articles identified from the exploratory search, we discerned 12 articles that were deemed relevant to this review. In the second step, we structured 3 blocks of keywords, each representing a facet of the RQs: physicians as the target population (block 1), patients’ health information behavior (block 2), and physicians’ perspectives or communication strategies with internet-informed patients (block 3; Textbox 1). Each block encompasses a set of synonyms and related Medical Subject Heading (MeSH) terms tailored to represent the aspect of the RQs under consideration. For instance, the patients’ health information behavior (block 2) includes terms like *internet-informed patients*, *health-related internet use*, e-patients, *internet information*, *eHealth*, *health information search*, *internet health information*, and *online health information*. Within each block, synonymous terms and related MeSH terms were amalgamated using the OR operator. The 3 blocks were then combined with the AND operator. We used truncations to widen our search and set abstract or title limiters to circumvent the retrieval of an overwhelming number of results in several databases. This combination of terms was used across several platforms: PubMed, Scopus, CINAHL, Communication and Mass Media Complete, and PsycINFO.

**Search terms.**

**Block 1: physicians**

physicians, doctors

**Block 2: patients’ health information behavior**

internet-informed patients, health-related internet use, e-patients, internet information, eHealth, health information search, internet health information, online health information

**Block 3: physicians’ perspectives or communication strategies with internet-informed patients**

doctor–patient relation, physician-patient relation, physician-patient communication, doctor–patient communication, experience, attitude, strategy, belief

The first search was conducted in August 2022, and we initially restricted our search to the time frame from January 1, 1990, to August 1, 2022. All search outcomes were cataloged in a data set for subsequent screening. Before this screening, we assessed the effectiveness of our search algorithm by seeking out the 12 articles initially pinpointed from the exploratory search. All 12 articles were successfully located via our search algorithm, affirming its adequacy. We then performed a second search on December 24, 2023, using the same combination of keywords in the 5 databases used for the first search. The second search was conducted to collect studies published after August 1, 2022, the cutoff date for the first search. Multimedia Appendix 1 provides a detailed overview of the search algorithm and the total number of results obtained from each database.

### Inclusion and Exclusion Criteria

We included empirical qualitative, quantitative, and mixed methods studies that assessed physicians’ perspectives on patients’ internet health information–seeking behavior and their communication with internet-informed patients. A *physician* is defined as a medical doctor, either a specialist or a general practitioner (GP). This review focuses on physicians attending to physical health conditions. Studies had to be published in a peer-reviewed journal after 1990 and be written in English. We chose 1990 as the starting year because public access to the internet was not available before then. We did not consider the impact factor of the journals or their peer-review process. If a study covered multiple populations, such as physicians, nurses, and patients, we only considered those presenting the results for physicians separately from other groups. Textbox 2 lists the inclusion and exclusion criteria.

**Inclusion and exclusion criteria.**

**Inclusion criteria**

- Study topic: physicians’ perspectives regarding patients’ internet health information–seeking behavior
- Study populations: medical doctors who are attending to physical health conditions
- Study type: empirical studies using qualitative, quantitative, or mixed methods
- Language: written in English
- Publication year: published after the year 1990

**Exclusion criteria**

- Study topic: physicians’ perspectives in contexts unrelated to patients’ internet health information seeking, such as telemedicine, digital devices, electronic health records, and physicians’ own internet information–seeking behavior and social media use
- Study populations: nonmedical doctors or psychiatrists specializing in areas other than physical health
- Study type: reviews, evidence syntheses, study protocols, reports, book chapters, or any forms of gray literature
- Language: written in languages other than English
- Publication year: published before the year 1990

### Studies Identification

The first author (QL) and a research assistant independently assessed the titles and abstracts of these articles based on the inclusion and exclusion criteria. Any disagreements in selection were discussed with another author until a consensus was reached. Full-text screenings were performed by 2 authors independently, and any discrepancies were resolved through discussion.

### Data Analysis

A thematic analysis was conducted to identify prominent themes and to systematically organize the literature under these themes. This method permits the combination of qualitative and quantitative evidence [34] and has been used in previous reviews concerning physicians’ internet health information needs and their role in cancer care [35,36]. Both authors (QL and PJS) of the present review analyzed data from the included studies and synthesized them into themes and subthemes. The qualitative results of the included articles were used to describe the themes by using specific quotations that offer direct evidence of physicians’ experiences and perspectives. Quantitative results from survey studies were used to validate and support the identification of themes. For instance, if a theme emerged from the qualitative data suggesting that “physicians perceive that patients’ internet health information seeking boosts patients’ confidence in participating in their healthcare,” quantitative data were used to indicate the percentage of physicians who echoed the perspective that internet health information seeking boosts patient confidence. Our analysis focused solely on the raw data and results of the included articles, extracting direct evidence without incorporating the interpretations or conclusions drawn by the authors of the included studies. We did not use specialized qualitative software for data analysis. Instead, we created 2 structured tables (Tables S1 and S2 in Multimedia Appendix 2 [22,29,37-56]) in Microsoft Word (Microsoft Corp) to systematically list, organize, and synthesize all themes and subthemes alongside their associated qualitative and quantitative evidence. This approach facilitated clear organization of qualitative quotations and the corresponding survey evidence.

### Quality Assessment

Two tools were used by the first author to evaluate the risk of bias in the included studies. The Critical Appraisal Skills Program (CASP) checklist assessed the quality of qualitative research, while a tool developed by Hoy et al [57] evaluated quantitative research. Both tools comprise 10 questions that assess the study’s objectives, methodology, sampling, ethical considerations, and other aspects. A quantitative study was considered to have a moderate risk of bias if it satisfied 4 or 5 out of the 10 criteria and a low risk of bias if it met 6 to 8 criteria. The CASP does not categorize qualitative research as “high,” “medium,” or “low” quality; instead, it appraises the strengths and limitations of its methodology. It has been widely used in health-related qualitative evidence syntheses [58].

## Results

### Overview of the Articles

**Figure 1:**
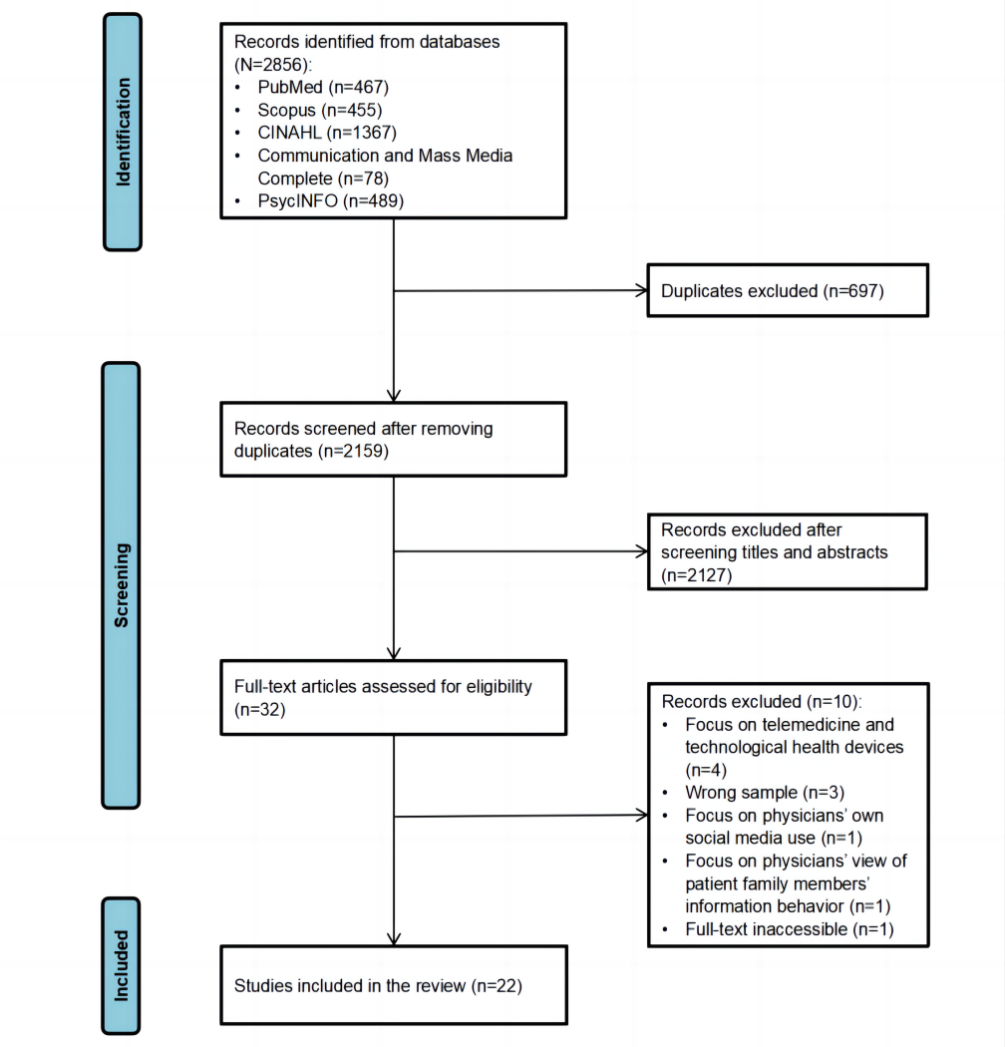
PRISMA (Preferred Reporting Items for Systematic Reviews and Meta-Analyses) flow diagram of the studies identified, screened, and assessed for eligibility.

Figure 1 illustrates the article selection process for this review in accordance with the PRISMA guidelines. Using the search algorithm described in the Search Strategy section, 2856 records were initially identified, which ultimately yielded 2159 (75.6%) unique articles after removing duplicates. After screening titles and abstracts, 32 (1.48%) articles remained for full-text assessment. In total, 10 (31%) articles that did not meet the inclusion criteria were excluded: 4 (40%) that focused on telemedicine and technological health devices; 3 (30%) with an incorrect sample (1 each targeting patients with cancer and caregivers, the general population, and nurses); 1 (10%) study that focused on physicians’ social media use; 1 (10%) study on physicians’ view of patient family members’ information seeking; and 1 (10%) study for which the full article was inaccessible. Ultimately, 22 (69%) articles were included in this review.

Of the 22 included articles, 11 (50%) used qualitative methods, including focus groups, interviews, and analysis of audio-recorded clinical consultations. Another 11 (50%) articles adopted a quantitative approach using survey data. Furthermore, 2 (18%) of the quantitative articles also incorporated a qualitative method through open-ended survey questions [37,38]. A total of 4 (18%) studies exclusively collected data from family physicians or GPs, 2 (9%) studies focused solely on oncologists, 1 (5%) study focused on dermatologists, while the remaining studies (n=15, 68%) encompassed physicians from various specialties. Most of the included studies were conducted in North America and Europe, encompassing the United States (n=4, 18%), Canada (n=2, 9%), Germany (n=4, 18%), the Netherlands (n=1, 5%), the United Kingdom (n=2, 9%), Switzerland (n=2, 9%), and Norway (n=1, 5%). Other countries featured in the studies included Israel (n=3, 14%), Oman (n=1, 5%), Brazil (n=1, 5%), and South Korea (n=1, 5%). One study interviewed physicians from 7 different countries: Australia, Israel, France, the United States, the Philippines, New Zealand, and Hungary. Table 1 provides an overview of the included articles.

**Table 1:** Overview of the included articles.

Study	Journal	Method	Data collection	Country	Participants
Wangler and Jansky [47], 2020	*Wiener Medizinische Wochenschrift*	Qualitative	Interviews	Germany	38 GPs^a^
Kim and Kim [42], 2009	*Informatics for Health and Social Care*	Quantitative	Web surveys	South Korea	430 specialists, 32 GPs, and 31 training doctors
Masters et al [49], 2020	*Journal of Medical Internet Research*	Quantitative	Surveys	Oman and Germany	108 surgeons
Ahmad et al [45], 2006	*Journal of Medical Internet Research*	Qualitative	Focus groups	Canada	48 family physicians
van Uden-Kraan et al [38], 2010	*Clinical Rheumatology*	Mixed methods	Surveys with open questions	The Netherlands	134 rheumatologists and 104 oncologists
Fujioka and Stewart [44], 2013	*Health Communication*	Quantitative	Web surveys	The United States	104 physicians
Murray et al [43], 2003	*Journal of Medical Internet Research*	Quantitative	Surveys	The United States	1050 physicians
Giveon et al [48], 2009	*Israel Medical Association Journal*	Quantitative	Surveys	Israel	118 family physicians
Caiata-Zufferey and Schulz [51], 2012	*Health Communication*	Qualitative	Interviews	Switzerland	17 physicians
Ahluwalia et al [40], 2010	*British Journal of General Practice*	Qualitative	Interviews	The United Kingdom	11 GPs
Moick and Terlutter [56], 2012	*Medicine 2.0*	Quantitative	Web surveys	Germany	287 physicians
Potts and Wyatt [54], 2002	*Journal of Medical Internet Research*	Quantitative	Web Surveys	The United Kingdom	748 physicians
Ohana and Barnoy [55], 2019	*Nursing Outlook*	Quantitative	Surveys	Israel	184 e-patients, 52 nurses, and 48 physicians^b^
Győrffy et al [39], 2020	*PLoS ONE*	Qualitative	Interviews	Australia, Israel, France, the United States, the Philippines, New Zealand, and Hungary	11 digitally engaged physicians
Shachar [29], 2022	*Social Science & Medicine*	Qualitative	Interviews	Israel	32 physicians
da Mota et al [50], 2018	*Revista da Associação Médica Brasileira*	Quantitative	Surveys	Brazil	183 physicians
Helft et al [37], 2003	*Journal of Clinical Oncology*	Mixed method	Surveys with open questions	The United States	266 oncologists
Sommerhalder et al [46], 2009	*Patient Education and Counseling*	Qualitative	Interviews	Switzerland	32 patients and 20 physicians^b^
Shen et al [22], 2015	*Psycho-Oncology*	Qualitative	21 audio-recorded clinical consultations	The United States	Oncologists (undefined number of physicians)
Fredriksen et al [41], 2018	*Health Care for Women International*	Qualitative	Interviews	Norway	13 Norwegian GPs, midwives, and physiotherapists^b^
MacDonald et al [52], 2018	*Journal of Medical Internet Research*	Qualitative	Interviews	Canada	12 physicians and nurses (3 GPs, 2 registered nurses, 1 nurse practitioner, 3 rheumatologists, 1 physician clinician-scientist, and 2 rheumatology fellows)^b^
Schick et al [53], 2023	*Journal of Medical Internet Research*	Qualitative	Interviews	Germany	16 patients and 12 dermatologists^b^

^a^GP: general practitioner.

^b^Only study results and quotations from physicians, including GPs and specialists, are considered.

In the quality assessment, all the qualitative studies (11/22, 50%) received “yes” responses to the first 5 questions on the CASP checklist. This indicates that these studies provided clear descriptions of their research aims and used appropriate methodologies, sampling procedures, and data collection techniques. Moreover, each study received positive responses to at least 8 out of the 10 questions. The primary area of concern, as denoted by negative responses, pertained to the following question on the checklist: “Has the relationship between the researcher and participants been adequately considered?” This suggests that many studies did not critically examine the researcher’s role, its associated potential bias, and influence during the research process. Furthermore, 2 (9%) articles that used mixed methods were evaluated with the tool for quantitative studies, as most of their results were presented numerically. Most of the included quantitative studies (7/11, 64%) exhibited a low risk of bias, while the remainder (4/11, 36%) displayed a moderate risk. Multimedia Appendix 3 [22,29,37-56] details the quality check for each study.

### Synthesis of the Studies

#### Overview of Categories

A total of 5 main categories were identified through the coding of evidence from the included articles. Two primary categories, “impacts on patients’ health and health management” and “Impacts on physician-patient relations and health care services,” represent physicians’ perceptions of patients’ internet health information seeking in terms of its effects on patients' health and health management, as well as on their relationships with physicians and on health care systems, respectively. These categories address RQ1. Two additional categories, “participative strategy” and “defensive strategy,” summarize a series of communication actions physicians take in response to internet-informed patients, thereby addressing RQ2. The final category, “physicians’ difficulties and needs,” addresses RQ3 by delving into the challenges and needs that physicians perceive when dealing with internet-informed patients. Multimedia Appendix 2 provides a detailed list of all subthemes, accompanied by their qualitative quotations and supporting quantitative survey evidence.

In the following sections, we first offer a brief description of each primary category, accompanied by its corresponding subthemes highlighted in italics. We then delve deeper into physicians’ perceptions, their choices regarding communicative strategies, and their identified difficulties and needs. Furthermore, we outline the antecedents of, or connections between, these categories. Direct qualitative quotations and survey evidence are provided to reinforce our observations.

#### Impacts on Patients’ Health and Health Management

Physicians believe that seeking health information on the internet can *inform/educate patients* about their health conditions and *enhance patients’ confidence* in participating in health care. The internet can also *provide social support* by connecting patients with their peers. However, physicians express concern that the internet might *misinform patients* with inaccurate information and some patients lack the capability to interpret internet information appropriately. Internet searches can potentially *trigger patients’ negative emotions*, such as anxiety or confusion. Moreover, physicians view *patients’ self-diagnosis and self-treatment* based on internet searches, conducted before medical consultations, in a negative light.

#### Impacts on Physician-Patient Relations and Health Care Services

Physicians believe that internet information seeking can *improve physician-patient relations,* as internet-informed patients are more empowered and informed in managing their health. Such searches also *encourage patients’ participation in decision-making*. Moreover, internet-informed patients can *improve the efficiency of medical consultations*, potentially shortening the duration when patients have already informed themselves about their conditions. However, there have been reports from physicians about *nonadherent patients* who disregard medical advice due to their internet searches. Some physicians sense *feelings of distrust* from internet-informed patients and feel that their medical *authority is being challenged* by them. They also sometimes experience *negative emotions,* such as anxiety and uneasiness, when interacting with internet-informed patients. Furthermore, physicians note *increased time and information demands*, as well as *inappropriate medical requests* made by internet-informed patients.

#### Participative Strategy

Physicians *appreciate internet searches* when patients bring internet information to consultations. They help patients *examine the information* and *acknowledge their limited expertise* on specific topics. They clearly *explain the diagnosis and treatment* plan to patients and strive to *understand patients’ emotional needs*, such as concerns about their health and motivations for conducting internet searches. Furthermore, physicians seek to *build ongoing relations with internet-informed patients* and *instruct patients on appropriate internet use*, including teaching them how to recognize appropriate information sources and recommending reliable internet sites.

#### Defensive Strategy

Physicians *decline to discuss* internet health information by displaying resistance or deferring patients to other specialists. They also *discredit the internet* as an unreliable information source and *devalue the internet health information* brought by patients.

#### Physicians’ Difficulties and Needs

Physicians face *extra responsibility* as information interpreters or examiners beyond their traditional professional practices, adding pressure on an already overwhelmed health system. Physicians desire *training to manage internet-informed patients, stay updated with technology,* and be informed about *reliable internet sites* that they can recommend to patients.

### Physicians’ Perceptions of Internet-Informed Patients

Diverse views on internet-informed patients existed among physicians. These views can be seen as a continuous spectrum of opinions ranging from positive to negative. In qualitative studies, a group of digitally engaged physicians who were active on social media generally held positive perceptions toward internet health information, regarding its impact on physician-patient communication and patients’ health [39]. In contrast, interviews with 11 GPs from the United Kingdom revealed generally negative perceptions [40]. Another interview study in Norway showed physicians with these 2 opposite opinions [41]. Between these 2 polarized views, a larger number of physicians expressed balanced perspectives on patients’ internet searches. In survey studies, many physicians held neutral opinions regarding the impact of internet searches on physician-patient relations [42,43]. These physicians recognized both the beneficial and detrimental effects of internet information on patients’ health [38,42,44]. Interview studies also showed that physicians’ views on internet-informed patients varied based on their interactions with individual patients [41,45]. In other words, they did not approach all patients with a singular attitude; rather, they often held more nuanced or balanced perceptions.

Physicians with positive perceptions valued the more equal physician-patient relationship fostered by internet-informed patients [39]. For instance, an interview study from Switzerland found that physicians with positive perceptions believed that internet-informed patients, by searching on the internet, become more informed about their health conditions and thus feel encouraged and more confident to engage collaboratively with their physicians in health care [46]. Similarly, physicians from Israel mentioned in interviews that internet-informed patients had assisted them in making diagnoses and pinpointing referrals, thereby making medical encounters more efficient [29]. The following quotations exemplify this positive perspective:

Because of the Internet, social media and technology, my patients were coming to me with more information and they weren’t looking to me to just solve a problem. They wanted to be involved in this problem. [39]

Male physiotherapist aged 36 years

I’d say spontaneously that it gives them [internet-informed patients] more right to have a say in a matter. They have, let’s say, more empowerment to join in the conversation. They then already have an opinion, and don’t come here thoroughly blank. [46]

Male physician, obtained medical degree in 1983

Physicians with negative perceptions expressed unpleasant feelings toward internet-informed patients. They felt that their authority was challenged by these patients [40]. Notably, 3 survey studies from South Korea, the United States, and the Netherlands showed that approximately 20% of physicians felt that internet-informed patients challenged or undermined their authority as medical professionals [38,42,43]. Furthermore, they perceived themselves as being devalued and distrusted by internet-informed patients [40,47]. Their views are expressed as follows:

For me that was the irritation, that the patient had far more trust in the computer and what they found on the web than in what I was trying to explain. [40]

female GP

I see a very big danger in the fact that the patient gets into a kind of tunnel through his/her constant search on the internet and then, in the end, is no longer receptive to the doctor’s advice. Again and again, I experience those patients who constantly feel misunderstood and do doctor hopping. [47]

female GP

In contrast, many physicians hold mixed perceptions of internet-informed patients, which are influenced by their interactions with individual patients. For instance, 2 qualitative studies conducted in Canada and Norway have revealed that physicians differentiate between patients who use the internet for self-education and those who use it for self-diagnosis and self-treatment [41,45]. Physicians tend to view patients who use the internet for self-education in a favorable light. These patients often bring internet information to the physician for confirmation and remain receptive to the physicians’ suggestions. In these cases, the internet serves as a helpful tool. In contrast, when patients use the internet to self-diagnose or self-treat, specifically, those who have already made up their minds before consulting a physician, they are often perceived as “challenging” patients. Physicians have reported feeling the need to defend their diagnosis or treatment plan, and this can evoke negative emotions, ranging from frustration to anger, when dealing with such self-diagnosing or self-treating patients [45]. Furthermore, physicians recognize the importance of established relationships with patients in shaping their perspectives. A positive prior relationship leads them to view the internet as beneficial [38]. Some physicians described it as follows:

I think there’s one situation where the Internet is useful. If the person has the diagnosis, and they want to find out more, educate themselves,... I find that’s actually helpful in cases where...it’s not time-consuming for me.

If they’re, however, using it to diagnose, then I think that’s where the problem lies... [45]

A focus group of family physicians

If the relationship is good, Internet use is not a problem. The biggest problem is with new patients with whom no relationship has yet been forged and who arrive with a certain assertivity or suspicion. [38]

Not available

Quantitative data also support the observation of physicians holding more neutral or balanced perceptions. A survey study of 406 US physicians who had previously encountered internet-informed patients during consultations found that 38% of the physicians consider the internet information brought by the internet-informed patients to benefit their relationship, 8% consider it harmful to the physician-patient relationship, and 54% provided neutral answers [43]. Another survey study involving 493 South Korean physicians found that 16.6% believed that discussing internet information with patients positively impacted their relationships. In contrast, 25.6% indicated that it had negative impacts, while 42.6% chose a neutral response, indicating that they perceive it as having no significant impact on the physician-patient relationship either way [42]. In alignment with these 2 studies, the proportion of physicians with varying perceptions do not show significant differences across other survey studies from the Middle East, South America, and Europe [38,48-50]. Overall, most physicians hold neutral perceptions.

In addition, physicians’ general perceptions of internet-informed patients appear to differ between different cultures, ages, and specializations. Focus groups involving 48 Canadian family physicians revealed that senior physicians felt a stronger sense of challenged authority than younger medical graduates [45]. In a survey of 108 surgeons, German surgeons were less content with internet information than their Omani counterparts [49]. Another survey from the Netherlands involving 238 oncologists and rheumatologists found that oncologists were less positive about internet use than rheumatologists [38]. However, these associations lack substantial evidence, as few studies have verified them.

### Physicians’ Communicative Strategies: How Do Physicians With Negative Perceptions Respond to Internet-Informed Patients?

Physicians’ communicative strategies were mostly observed in qualitative studies. When physicians develop negative perceptions, they are likely to adopt specific actions or strategies aligning with these perceptions. For instance, an interview study with 38 GPs revealed that physicians who viewed themselves as the primary “decision-making and instructing authority” actively discouraged patients from internet searches, believing that other sources of information that might conflict with their own should be eliminated. In contrast, GPs who did not emphasize their authoritative role chose to collaborate with internet-informed patients, recommending reliable internet information sites and jointly examining internet information without seeking to prevent patients from further internet search [47].

Being identified in several studies [45,47], defensive actions have been termed by scholars as *resistance* [51] or *negative mediation* [44]. Defensive actions can be categorized into 2 types. The first type involves refusing to discuss internet information, which can manifest as resistance to discussing such information, terminating the physician-patient relationship by referring patients to other specialists, or even suggesting an additional charge for discussing internet-based information. The second type is about devaluing internet health information. This includes actions like discrediting the internet as a source, devaluing the health information that patients obtain from the internet, and correcting patients’ misbeliefs with the implication that they should stop searching on the internet for health information [41,44,45,47,51]. Some physicians described this approach as follows:

When patients tell me, “yes, but on the Internet,”... I always cut short: “On the Web you find everything and its opposite, so forget it all and listen to what I’m saying, which is the standard.” [51]

Male gynecologist aged 63 years

If they come in and it’s too much and it’s too specialized.... I let them slug it out with the specialist. They’re paid very special money to do this kind of work. [45]

A focus group of family physicians

Physicians have claimed to take defensive actions to reduce the risk of internet health information for their patients [51]. A survey of 104 American physicians indeed showed that their negative assessment of the quality of internet information was positively correlated with more defensive actions [44]. However, another survey study of 1050 American physicians found that their perception of the potential harm of internet information to health was positively associated with their feelings of challenged authority [43]. Hence, the defensive strategy can be seen as coping mechanism that helps physicians defend themselves from the challenges and potential emotional unpleasantness posed by internet-informed patients.

### Physicians’ Communicative Strategies: How Do Physicians Collaborate With Internet-Informed Patients?

Physicians who have a positive perception of their patients’ internet searches tend to work with their patients more collaboratively [44,51,52]. Their actions with internet-informed patients promote physician-patient relations by making the best use of the internet. These physicians believed that patients can also provide information and negotiate health care decisions, and they acknowledged the value of internet information brought by patients to the consultation [29]. As stated by the physicians, they would show interest in the information that patients bring, make patients feel respected and listened to, and examine patients’ information. In addition, they believed that recommending reliable internet health information sites is an excellent way to guide patients in using internet information more effectively [39,52,53]. They also emphasized that the internet will never replace the human touch that physicians offer. They claimed to provide internet-informed patients with holistic care and to build ongoing relationships based on trust and familiarity [29].

While participative physicians acknowledged the risks associated with internet information, they regarded the internet as a powerful and legitimate health information source. Thus, they saw it as an opportunity for patients and believed that physicians should guide them in using this medium appropriately [51]. These actions can be categorized as the participative strategy, as opposed to the defensive one. Physicians take participative actions to “join in” with internet-informed patients in a way that allows them to guide and help patients navigate the internet with physicians’ professional expertise. The participative approach is described as follows:

Yeah for me, for instance, the use of sites, I know patients when they come to you and you have to provide information they usually get shocked first to get a diagnosis and second to start treatment. And so I give them readings. I print some information for them and tell them if they have more questions to go to these sites and then you come back with me and we can discuss it if you want. [52]

Male physician and scientist, 29 years in practice

Often people find information you don’t have. Why? We’re doctors, we’re constantly being updated, [but] we’re not up to date on everything. We don’t know everything about everything.... I actually like it when someone comes and tells me something I know nothing about, and I leave his room, and I go straight to the computer...and start reading about what they said. And if it’s relevant, I can sometimes find myself incorporating [it] into my work. Definitely.... They come, they tell you something, I’ve never heard about it, I’m willing to check. And then after you check, you become a slightly better doctor. Because you know more. [29]

Cardiologist

Apart from physicians’ self-claims, 1 study analyzed audio-recorded real clinical consultations between oncologists and patients with breast cancer in Switzerland [22]. The authors observed a series of participative actions with internet-informed patients, including physicians encouraging patients to use the internet, acknowledging the internet as an information tool, helping patients identify reliable internet information sources, providing detailed information to clarify patients’ internet information, and admitting their limited knowledge on the topic being discussed. These actions align closely with the participative approaches that physicians claimed in other studies [22].

Similar to physicians' mixed perceptions of internet-informed patients, some physicians have reported shifting between different communication strategies. An interview study with 17 Swiss physicians revealed that they would shift between participative and defensive strategies depending on their interactions with individual patients [51]. They tended to be cautious toward internet information when the patients demonstrated a low level of health literacy, as judged by the patient’s or the family member’s education level and the quality of information that the patient had brought. Furthermore, patients’ disrespect for physicians’ expertise and their reluctance to consider physicians’ suggestions also prompted defensive actions from physicians [51]. This characterization of patients who provoke a defensive response coincides with the description of self-diagnosing or self-treating patients given by physicians who maintain more neutral or balanced perceptions regarding patients’ internet searches. Physicians’ communication strategies, much like their perceptions, are not strictly limited to being participative or defensive. They adapt their communication approaches to suit individual patients. One physician illustrated his adaptability between different patients as follows:

You need to do the right thing with the right person. With some people you take the time to look at the information together, to evaluate it together. But there are also situations where you say no, I don’t want to go into it. You have to consider, evaluate and grade, you need to weed some things out and to keep others...

A few times, when I was really exasperated, I have said: “Time is up!” Those people were arrogant, and I have said “Time is up!” Two of them then left really pissed, but after the third repetition of the same thing.... Some patients do not want to understand: They have their idea and they want me to agree with it. [51]

Male urologist, aged 53 years

### Physicians’ Difficulties and Needs

With the introduction of internet information to medical consultations, physicians have frequently expressed in interview studies that their traditional roles have expanded to include responsibilities as internet information interpreters or examiners [39,41,45,46,52,53]. Two survey studies indicated that physicians believed patients brought internet information to consultations largely to seek physicians’ opinions on that information [42,43]. Internet-informed patients are typically perceived as more demanding in terms of information and time compared with regular patients [49,54,55]. Furthermore, while physicians with negative views of internet-informed patients are often reluctant to assume this new responsibility [45], even proactive physicians have expressed time constraints when addressing internet-informed patients within an already burdened health system [39].

In addition, some physicians highlighted challenges in dealing with extreme cases [47]. Interviews with UK family physicians revealed feelings of being “left alone” when dealing with patients who exhibit intense health anxiety and excessive internet information searches. These physicians felt ill-equipped, both in terms of psychological expertise and time, to handle such situations. They favored the development of structured training that aids in identifying such extreme cases. Another group of digitally engaged physicians expressed a need for an updated medical curriculum to assist them in better establishing relations with internet-informed patients [39]. However, the studies included in this review provide limited details on the specific training needs of physicians, preventing a detailed exploration of their preferred curriculum.

Several studies highlighted the physicians’ desire to stay updated with emerging technologies and trustworthy internet information sources for patient reference [38,45,47,48]. For example, a survey study of 118 family physicians revealed that 58% saw a need for training on internet use [48]. In another mixed methods study, 53% of physicians expressed difficulty in staying informed about credible internet health sites [38]. One physician illustrated it as follows:

It is imperative that doctors are trained in internet usage. I rarely know which website to recommend to patients. [38]

Not available

## Discussion

### Principal Findings

This review has examined physicians’ perceptions of patients’ internet health information–seeking behavior and their communication experiences with internet-informed patients. We included a mix of qualitative and quantitative studies, which enabled us to gain a deeper understanding from physicians’ perspectives and explore connections between physicians’ perceptions and their communicative approaches.

We first identified a diversity of perceptions among physicians regarding patients’ internet searches. These perceptions can be viewed as a continuous spectrum of opinions ranging from positive to negative. While some physicians hold more positive or negative views on patients’ internet searches, a greater number of physicians expressed balanced views toward internet-informed patients. They acknowledge the benefits of internet health information but also raise concerns. The findings indicate that physicians, in general, are not entirely resistant to interacting with internet-informed patients. Their potential in educating and empowering patients to manage their health in this digital age should be further investigated and emphasized. For instance, scholars have drawn attention to the role of physicians in addressing internet misinformation. They have argued that although laypeople lack the medical expertise to distinguish the quality of internet material, the internet can become an extremely helpful tool with the collaboration of physicians [17].

Discrepancies exist when comparing the perceptions of physicians and patients. Previous reviews have concluded that patients tend to believe that internet searches make their consultations with physicians more effective [13]. However, our review found that physicians generally perceive their experiences with internet-informed patients as more time-consuming and demanding [42]. Physicians are also commonly concerned about the risk of misinformed patients [52,54]. Their concerns are not only about the poor quality of internet information but also about the patient’s capability to process a large volume of information. The divergences between patients’ and physicians’ perspectives highlight the importance of understanding both parties’ viewpoints. Future policy makers and researchers should consider these differences while investigating internet use and physician-patient communication.

We also identified 2 types of communicative strategies that physicians adopted to deal with internet-informed patients. These strategies include the defensive approach and the participative approach, each containing a series of specific communicative actions. Physicians’ perceptions are linked to their strategies: positive perceptions toward internet-informed patients lead to a participative approach, while negative perceptions lead to a defensive approach. The 2 strategies were primarily identified through interview studies with physicians and are, therefore, based on their self-claims. Future research should build on our findings and delve into the interaction process between physicians and patients. This exploration will provide a better understanding of how physicians implement these strategies in actual medical consultations and how various strategies impact both the patients’ use of internet material and the physician-patient relationship.

Furthermore, physicians shift between the 2 strategies based on their interaction with specific patients. The identification of neutral physicians and physicians’ shift between the 2 communicative strategies emphasizes the dyadic nature of physician-patient relations. On the one hand, patients are cautious about disclosing internet-sourced information to their physicians and constantly evaluate the possible reactions of physicians to their information [23,25]. On the other hand, physicians adopt different attitudes and communicative approaches based on their interaction with that particular patient [51]. The communication process between the patient and the physician plays an essential role in determining whether patients will introduce internet information and how the information will be responded to by the physician during a consultation.

This study also identified physicians’ difficulties and needs with internet-informed patients. The most common difficulty is the time pressure to address information and time demands from internet-informed patients. The medical systems in many countries are already overwhelmed [59]; therefore, the time issue could be difficult to address. Other common needs include training on new technologies and being informed of reliable internet sites that can be recommended to patients [38,48,53]. Some physicians also raised a need for training to help them address extreme cases of patient internet searches and form a more satisfying relationship with internet-informed patients [39,47]. This finding provides new insights into the medical teaching curriculum. Introducing special training that showcases trustworthy medical websites recommended for patients to reference could better equip physicians for medical encounters with internet-informed patients.

However, it is important to note that these training needs were primarily demonstrated in studies where physicians held more positive views of the internet [38,39,48]. Therefore, the expressed training needs primarily come from physicians who are receptive to internet information and tend to adopt a participative strategy toward internet-informed patients. Their motivation for such training programs likely revolves around enhancing their collaborative skills with internet-informed patients. Future research should take into account the perspectives of physicians with negative views, as they might have different concerns and might not be in favor of training specifically designed to enhance communication with internet-informed patients. Nonetheless, it is worth noting that newer medical graduates are more receptive to internet-informed patients compared with senior physicians [45]. Some medical educators have considered implementing communication training to prepare medical students for future consultations with internet-informed patients [60], and it has been observed that such training boosts the competency of medical students [61].

### Limitations

Our review has certain limitations. First, the specialization of physicians may influence their perspectives on internet-informed patients; however, we could not draw concrete conclusions regarding the differences among medical specialists, as only a few of the included studies had examined them. In addition, some included studies focused solely on GPs or oncologists, which might skew the prevalence of their perspectives in this review and potentially diminish the applicability of our results to other specialists.

Second, most of the included studies are from Europe and North America, with few focusing on physicians from Eastern countries, such as South Korea and Israel. This makes it challenging to draw further comparisons between different cultures. Particularly, in some East Asian cultures dominated by Confucianism, strong hierarchies between physicians and patients still exist [62]. The impact of internet information on changing physician-patient relationships in these cultures was found to be limited [24]. Physicians from such Eastern cultures might exhibit different perceptions and communicative actions toward internet-informed patients compared with their Western counterparts, where the concept of an equal physician-patient relationship is more common.

Third, the data used to observe communicative strategies primarily originated from qualitative interviews with physicians. Using quantitative data and observations from real-life consultations could strengthen our results, which are currently based on physicians’ self-claims.

### Conclusions

This review underscores the varied perceptions physicians hold toward internet-informed patients. Physicians’ choice of communication strategies, whether adopting a participative or defensive approach, is intricately linked to their perceptions and their interactions with individual patients. Incorporating a medical teaching curriculum that introduces reliable internet sites to physicians for patient reference and provides updates on technology can potentially assist physicians in better coping with internet-informed patients.

## Appendix

Multimedia Appendix 1 Keyword combinations and databases.

Multimedia Appendix 2 Description of themes and supporting evidence.

Multimedia Appendix 3 Quality assessment.

Multimedia Appendix 4 PRISMA checklist.

## Abbreviations

CASP: Critical Appraisal Skills Program
GP: general practitioner
MeSH: Medical Subject Headings
PRISMA: Preferred Reporting Items for Systematic Reviews and Meta-Analyses
RQ: research question
