# Supplementary material for: Physician Perspectives on Internet-Informed Patients: Systematic Review
Source: J Med Internet Res. 2024 Jun 6;26:e47620. doi: 10.2196/47620 (PMC11190621; doi:10.2196/47620)
Supplement: Multimedia Appendix 1 [file jmir_v26i1e47620_app1.docx]

**Appendix 1. Keywords combination and databases**

| Databases | Keywords combination | Results |
| --- | --- | --- |
| **CINAHL (EBSCO)** | (physicians OR doctors) AND ("internet-informed patients” OR "health-related Internet use" OR e-patients OR "Internet information" OR eHealth OR "health information search*" OR "Internet health information" OR "online health information”) AND ("doctor–patient relation*" OR "physician-patient relation*" OR "physician-patient communication" OR "doctor-patient communication" OR experience OR attitude OR strateg OR belief) | **1367** |
| **Communication and Mass Media Complete (EBSCO)** | (physicians OR doctors) AND ("internet-informed patients” OR "health-related Internet use" OR e-patients OR "Internet information" OR eHealth OR "health information search*" OR "Internet health information" OR "online health information”) AND ("doctor–patient relation*" OR "physician-patient relation*" OR "physician-patient communication" OR "doctor-patient communication" OR experience OR attitude OR strategy OR belief) | **78** |
| **PsycINFO (EBSCO)** | (physicians OR doctors) AND ("internet-informed patients” OR "health-related Internet use" OR e-patients OR "Internet information" OR eHealth OR "health information search*" OR "Internet health information" OR "online health information”) AND ("doctor–patient relation*" OR "physician-patient relation*" OR "physician-patient communication" OR "doctor-patient communication" OR experience OR attitude OR strategy OR belief) | **489** |
| **PubMed** | (physicians[Title/Abstract] OR doctors[Title/Abstract] OR Physicians[MeSH Terms]) AND ("internet-informed patients"[Title/Abstract] OR "health-related Internet use"[Title/Abstract] OR e-patients[Title/Abstract] OR "Internet information"[Title/Abstract] OR eHealth[Title/Abstract] OR "health information search*"[Title/Abstract] OR "Internet health information"[Title/Abstract] OR "online health information"[Title/Abstract]) AND ("doctor–patient relation* "[Title/Abstract] OR "physician-patient relation*"[Title/Abstract] OR "physician-patient communication"[Title/Abstract] OR "doctor-patient communication"[Title/Abstract] OR Physician-Patient Relations[MeSH Terms] OR experience[Title/Abstract] OR attitude[Title/Abstract] OR strategy[Title/Abstract] OR belief[Title/Abstract]) | **467** |
| **Scopus** | ( TITLE-ABS ( physicians ) OR TITLE-ABS ( doctors ) ) AND ( TITLE-ABS ( "internet-informed patients" ) OR TITLE-ABS ( "health-related Internet use" ) OR TITLE-ABS ( e-patients ) OR TITLE-ABS ( "Internet information" ) OR TITLE-ABS ( ehealth ) OR TITLE-ABS ( "health information search*" ) OR TITLE-ABS ( "Internet health information" ) OR TITLE-ABS ( "online health information" ) ) AND ( TITLE-ABS ( "doctor–patient relation*" ) OR TITLE-ABS ( "physician-patient relation* " ) OR TITLE-ABS ( “physician-patient communication” ) OR TITLE-ABS ( "doctor-patient communication" ) OR TITLE-ABS ( experience ) OR TITLE-ABS ( attitude ) OR TITLE-ABS ( strategy ) OR TITLE-ABS ( belief ) ) | **455** |
