# Supplementary material for: Physician Perspectives on Internet-Informed Patients: Systematic Review
Source: J Med Internet Res. 2024 Jun 6;26:e47620. doi: 10.2196/47620 (PMC11190621; doi:10.2196/47620)
Supplement: Multimedia Appendix 2 [file jmir_v26i1e47620_app2.docx]

**Appendix 2. Description of themes and supporting evidence**

**Table S1. Results of Physicians’ perceptions of Internet-Informed Patients (IIP)**

| **Themes** | | **Description with qualitative evidence** | **Supporting survey results** |
| --- | --- | --- | --- |
| **Impacts on patients’ health and health** **management.** | | | |
| *Positive* | | | |
|  | Inform/educate patients | Patients use internet health information to inform/educate themselves about their health condition (Ahmad et al., 2006; Fredriksen et al., 2018; Helft et al., 2003; MacDonald et al., 2018; Schick et al., 2023; Sommerhalder et al., 2009).  *There is a benefit to having patients who are well-informed; one can assume that certain things are known. One doesn’t have to start the diagnosis or the therapy from scratch. One can discuss the treatment better. That’s certainly an advantage.* [Male, medical degree in 1989] (Sommerhalder et al., 2009).  *I think there’s one situation where the Internet is useful. If the person has the diagnosis, and they want to find out more, educate themselves…, I find that’s actually helpful in cases where…it’s not time-consuming for me* [A focus group of family doctors] (Ahmad et al., 2006). | - 50.1% of physicians agreed or strongly agreed that internet health information improves patients’ understanding of medical conditions and treatment, and 34.5% gave neutral responses, 5-point Likert scale (1=strongly disagree, 5=strongly agree), N=493 (Kim & Kim, 2009). - More than half of physicians indicated that IIP are often better informed about their illness (54% of participants) and treatment options (51% of participants), 5-point Likert scale (1=almost never, 5=almost always), 236≤N≤238 (Van Uden-Kraan et al., 2010). - Physicians generally agreed that internet use increase patients’ knowledge and awareness of health issues (M=3.76, SD=0.75), 5-point Likert scale (1=strongly disagree, 5=strongly agree), N=90 (Fujioka & Stewart, 2013). |
|  | Enhance patients’ confidence | Patients are more confident in participating in their healthcare (Sommerhalder et al., 2009).  *I’d say spontaneously that it gives them [Internet-informed patients] more right to have a say in a matter. They have, let’s say, more empowerment to join in the conversation. They then already have an opinion, and don’t come here thoroughly blank.* [Male, medical degree in 1983] (Sommerhalder et al., 2009). | - 44.8% of physicians agreed or strongly agreed that exposure to personally relevant internet health information increases the patient’s sense of confidence and 28.6% of physicians gave neutral responses, 5-point Likert scale (1=strongly disagree, 5=strongly agree), N=493 (Kim & Kim, 2009). - 57% of the physicians indicated that patients often become more assertive due to health-related internet use, 5-point Likert scale (1=almost never, 5=almost always), 236≤N≤238 (Van Uden-Kraan et al., 2010). |
|  | Provide patients with social support | Patients gain social support from other patients on the Internet (MacDonald et al., 2018; Schick et al., 2023).  *I think it allows people to not feel alone, to be able to more easily connect with other patients...It’s sort of like the strength in feeling like you’re part of a community as opposed to be isolated that I think empowers people to be able to do that.* [Rheumatologist, female, 25 years in practice] (MacDonald et al., 2018). | - Physicians slightly agreed that internet use helps patients obtain social support (M=3.32, SD=0.95) and communicate with other patients (M=3.22, SD=0.90). 5-point Likert scale (1=strongly disagree, 5=strongly agree), N=90 (Fujioka & Stewart, 2013). |
| *Negative* | | | |
|  | Misinform patients | Patients are misinformed by inaccurate internet health information or inappropriate interpretation of internet health information (Ahmad et al., 2006; Fredriksen et al., 2018; Schick et al., 2023).  *They [patients] are getting full of rather stupid facts in many cases, which they do not know how to interpret, which are usually misinformation.* [A focus group of family physicians] (Ahmad et al., 2006). | - 64.9% of physicians disagreed or strongly disagreed that internet health information is accurate in general, and more physicians (85.4%) disagreed or strongly disagreed that most patients can judge the relevance of internet health information for their conditions, 5-point Likert scale (1=strongly disagree, 5=strongly agree). In addition, 70.4% of physicians believed that the internet health information brought by IIP was irrelevant to their health conditions, a 5-point Likert scale (1=not at all relevant, 5=very relevant), N=493 (Kim & Kim, 2009). - Physicians indicated that patients sometime (70%) or often (17%) draw wrong conclusions about their illness based on internet health information, 5-point Likert scale (1=almost never, 5=almost always), 236≤N≤238 (Van Uden-Kraan et al., 2010). - Physicians agreed that there is a risk of obtaining misinformation on the internet (M=3.95, SD=0.75) and misinformed patients (M=3.79, SD=0.89). 5-point Likert scale (1=strongly disagree, 5=strongly agree), N=91 (Fujioka & Stewart, 2013). - 84% of physicians rated their patients as only fair or poor (rather than good, very good, or excellent) at appraising the quality of internet health information, 5-point Likert scale (1=poor, 7=excellent), N=1050 physicians (Murray et al., 2003). - Physicians generally agreed that patients can misunderstood health-related Internet health information or are unable to differentiate between accurate and inaccurate content (M=5.53, SD is not available), 7-point Likert scale (1=strongly disagree, 7=strongly agree), N=287 (Moick & Terlutter, 2012). - Physicians disagreed that most patients know which online health information is reliable or not (M=1.61, SD is not available), 5-point Likert scale (1=totally disagree, 5=totally agree), N=183 (da Mota et al., 2018). |
|  | Trigger patients’ negative emotions | Patients show a series of negative emotions, including confusion, unnecessary fear and anxiety, due to internet search (Ahluwalia et al., 2010; Ahmad et al., 2006; Helft et al., 2003; MacDonald et al., 2018; Wangler & Jansky, 2020).  *They are bringing up sort of obscure articles and stuff about different conditions, and some of them are pretty scary.…They think everything is happening.* [A focus group of family physicians] (Ahmad et al., 2006).  *Excessive Internet searches often do not contribute to a better understanding but increase the patients’ need for discussion and clarification because people are massively confused and unsettled. You can see that very well when you look at how many people come to my office hours.* [GP, female] (Wangler & Jansky, 2020). | - 74.5% of physicians agreed or strongly agreed that internet health information promotes patients’ unnecessary fear or concern about their health, 5-point Likert scale (1=strongly disagree, 5=strongly agree), N=493 (Kim & Kim, 2009). - Physicians slightly agreed that online information results in unnecessary fear over health in patients (M=3.33, SD is not available), 5-point Likert scale (1=totally disagree, 5=totally agree), N=183 (da Mota et al., 2018). - Most physicians indicated that patients are often (32%) or sometimes (53%) unnecessarily concerned due to internet health information, 5-point Likert scale (1=almost never, 5=almost always), 236≤N≤238 (Van Uden-Kraan et al., 2010). - Physicians somewhat agreed that internet searches increase patients’ anxiety and health concerns (M=3.75, SD=0.84). 5-point Likert scale (1=strongly disagree, 5=strongly agree), N=91 (Fujioka & Stewart, 2013). |
|  | Patients’ self-diagnosis/treatment | Patient had decided their diagnoses/treatment based on internet health information before meeting physicians (Ahmad et al., 2006; Fredriksen et al., 2018; Van Uden-Kraan et al., 2010; Wangler & Jansky, 2020).  *Recently, I saw a woman with fibromyalgia and her partner who didn’t want to accept it. He found information on the Internet stating that the symptoms of FM can be the same as with a vitamin B12 deficiency. They refused to see that a vitamin B12 deficiency was out of the question here. So in this case the information from the Internet was used to prove that it is something other than FM, and that’s when it becomes difficult.* [Not available] (Van Uden-Kraan et al., 2010). | - 12% of physicians believed that internet use leads patients to order dangerous or ineffective drugs or other health products, N=375 (Potts & Wyatt, 2002). |
| **Impacts on physician-patient relation / healthcare services.** | | | |
| *Positive* | | | |
|  | Improve physician-patient relation | Internet health information improves physician-patient relation (Győrffy et al., 2020; Helft et al., 2003; MacDonald et al., 2018).  *… I think if we have an engaged patient then we empower them to be able to really manage their disease a whole lot more efficiently. So I think we’ll have better outcomes because we’ll have better care that overall fits better with the patient. So that to me is, we’ll have a better relationship with the patient, we’ll have better management of things other than just the medications and the medical aspects of the disease, which is really important for the quality of life and the dealing with the person as a whole and the disability of the whole*. [Rheumatologist, female, 25 years in practice] (MacDonald et al., 2018).  *I think that the technology should empower this relationship rather than just take it away.* [Pediatrician, female] (Győrffy et al., 2020). | - Physicians slightly agreed that communication with IIP would lead to an improvement in the physician-patient (M=4.63, SD=1.9), 7-point Likert scale (1=strongly disagree, 7=strongly agree), N=108 surgeons (Masters et al., 2020). - Most physicians believed that the internet health information brought by patients to the consultation had a beneficial (38%) or neutral (54%) effect on the physician-patient relationship, 3-point scale (1=improved, 2=no difference, 3=worsened), N=406 physicians who encountered an IIP in their last medical consultation (Murray et al., 2003). - Physicians had neutral views on the jeopardize of online health information on good physician-patient relationship (M=2.98, SD is not available), 5-point Likert scale (1=totally disagree, 5=totally agree), N=183 (da Mota et al., 2018). - 42.6% of physicians felt the discussion of internet health information has a neutral impact on physician-patient relations, 16.6% felt a positive impact, and 25.6% felt a negative impact N=493 (Kim & Kim, 2009). - Physicians slightly agreed that online information seeking improves physician-patient relationship (M=4.24, SD is not available), 7-point Likert scale (1=strongly disagree, 7=strongly agree), N=287 (Moick & Terlutter, 2012). - Physicians slightly disagreed that internet searches decrease the quality of their interaction with the patients (M=2.75, SD=0.93), 5-point Likert scale (1=strongly disagree, 5=strongly agree), N=91 (Fujioka & Stewart, 2013). |
|  | Encourage patients’ participation in decision-making | Patients are more engaged in making health decisions with the physicians (Győrffy et al., 2020; MacDonald et al., 2018; Sommerhalder et al., 2009).  *[The Internet-informed patients] are more responsible. They are well-informed. They don’t accept just anything the doctor says. They ask. They are more in charge of themselves.* [Male, medical degree in 1975] (Sommerhalder et al., 2009).  *Because of the Internet, social media and technology, my patients were coming to me with more information and they weren't looking to me to just solve a problem. They wanted to be involved in this problem.* [Physiotherapist, male, 36 years old] (Győrffy et al., 2020). | - Physicians indicated that IIP somewhat often have higher expectations to be involved in their treatment decision making (M=3.79, SD=0.98), 5-point Likert scale (1=never, 5=always), N=48 (Ohana & Barnoy, 2019). - 78% of physicians believed that IIP sometimes or very often become more able to participate in the decision-making process about their treatment. In addition, rheumatologists (M=3.2, SD=0.71) showed stronger sense of improved participation than oncologists (M=3.0, SD=0.77), 5-point Likert scale (1=almost never, 5=nearly always), 224≤N≤237 (Van Uden-Kraan et al., 2010). |
|  | Improve efficiency of medical consultation | The efficiency of medical consultations is improved (Ahmad et al., 2006; Schick et al., 2023; Shachar, 2022; Van Uden-Kraan et al., 2010).  *Using the Internet also yields a shorter duration of the consultation because patients are better informed about their illness and need less explanation.* [Not available] (Van Uden-Kraan et al., 2010). | - 16% of physicians believed that patients bringing internet health information to the consultation improved time efficiency, while only 38% believed that it had worsened it and 45% of physicians gave neutral responses. 3-point Likert scale (1=improved, 2=no difference, 3=worsened), N=408 physicians who encountered an IIP in their last medical visit (Murray et al., 2003). - 13.6% of physicians felt that discussion on patient’ internet health information enhances the time efficiency, while 40.2% felt that it serves as a hindrance and 30.4% gave neutral responses, N=493 (Kim & Kim, 2009). |
| *Negative* | | | |
|  | Non-adherent patients | Patients become less adherent to physicians’ medical advice (Wangler & Jansky, 2020).  *I see a very big danger in the fact that the patient gets into a kind of tunnel through his/her constant search on the Internet and then, in the end, is no longer receptive to the doctor’s advice. Again and again, I experience those patients who constantly feel misunderstood and do doctor hopping.* [GP, female] (Wangler & Jansky, 2020). | - 59.0 % of physicians reported that they had ever encountered patients who changed recommended treatment after having seen internet health information, N=183 (da Mota et al., 2018). - 54.8% of physicians disagreed or strongly disagreed that internet health information encourages patients to follow treatment instructions or advice from their physicians, 5-point Likert scale (1=strongly disagree, 5=strongly agree), N=493 (Kim & Kim, 2009). |
|  | Physicians’ perceived distrusts | Physicians perceive distrust from IIP (Ahluwalia et al., 2010; Ahmad et al., 2006; Sommerhalder et al., 2009; Wangler & Jansky, 2020).  *For me that was the irritation, that the patient had far more trust in the computer and what they found on the web than in what I was trying to explain.* [GP, female] (Ahluwalia et al., 2010).  *Yes, it seems that [the Internet-informed patients] are those who believe less in doctors, also it requires more time to explain to them that they misunderstood what they read on the Internet*. [Male, medical degree in 1975] (Sommerhalder et al., 2009). | - Physicians slightly disagreed that internet use decreases patients’ trust in medical professionals (M=2.78, SD=1.04), 5-point Likert scale (1=strongly disagree, 5=strongly agree), N=91 (Fujioka & Stewart, 2013). |
|  | Physicians’ challenged authority | Physicians feel that their authority was challenged by IIP (Ahluwalia et al., 2010; Sommerhalder et al., 2009).  *That’s what I’ve been experiencing by now for the last 20 years; my professional authority isn’t as sacred as it used to be. I can’t say anymore that’s it, that’s what I see, this is what we know and the patients are trusting and believe that we know best. It’s no longer like this.* [Female physician, medical degree in 1978] (Sommerhalder et al., 2009). | - 20.9% of physicians felt that the IIP was challenging their authority, N=493 (Kim & Kim, 2009). - Physicians disagreed that they feel they might lose authority and control with IIP (M=2.82, SD=1.7), 7-point Likert scale (1=strongly disagree, 7=strongly agree). In addition, German surgeons felt more loss of authority and control than Omani surgeons, t (106) = −3.53, P =.001, N=108 surgeons (Masters et al., 2020). - 17% of physicians felt that the IIP was challenging their authority, N=406 physicians who encountered an IIP in their last medical visit (Murray et al., 2003). - Physicians generally disagreed that they have lost authority and control when facing IIP (M=2.78, SD is not available), 7-point Likert scale (1=strongly disagree, 7=strongly agree), N=287 (Moick & Terlutter, 2012). - Physicians indicated that Internet use sometimes (22% of physicians) or often (6%) undermines their authority, 5-point Likert scale (1=almost never, 5=almost always), 236≤N≤238 (Van Uden-Kraan et al., 2010). |
|  | Physicians’ negative emotions | Physicians have unpleasant emotions (e.g., anxious, uneasy) when facing IIP (Ahluwalia et al., 2010; Ahmad et al., 2006; Wangler & Jansky, 2020).  *Her (the patient) feelings of anxiety affected me and I began to feel under pressure and anxious as well ...* [GP, female] (Ahluwalia et al., 2010).  *“... this is what I want, give it to me”, so it felt quite aggressive and it made me feel defensive and that automatically made me feel on the back foot and out of control actually.* [GP, female] (Ahluwalia et al., 2010). | - 23% of physicians agreed or totally agreed that they feel uneasy when patients present them with internet health information, 4-point Likert scale (1=totally disagree, 4=totally agree), N=116 (Giveon et al., 2009). - 9% of oncologists reported that they sometimes or always felt threatened when patients brought Internet health information to the consultation, while 90% of oncologists reported that they rarely or never felt threatened with IIP. Additionally, oncologists who reported difficulty discussing internet health information were more likely to feel threatened by IIP (Pearson correlation coefficient is not available, P<.001), N=266 (Helft et al., 2003). |
|  | Time and information demand | Physicians perceived more time or information demand from IIP (Ahmad et al., 2006; Fredriksen et al., 2018; Győrffy et al., 2020; Schick et al., 2023; Wangler & Jansky, 2020).  *I do not mind patients coming in with information, but it is very hard if they present you with a package of, you know, 60 sheets.... Time is really at a premium, so it makes it very difficult.* [A focus group of family physicians] (Ahmad et al., 2006).  *The expectations from people in our community for their health care is much higher than they were when I first graduated over 20 years ago. So, when I first graduated people wanted some information but not a lot. And many people didn't really want much information at all. But now many people want a lot more information and the expectations for positive outcomes are much higher.* [Rheumatologist, female] (Győrffy et al., 2020). | - Physicians slightly agreed that consultations with IIP are more time-consuming than with normal patients (M=4.45, SD=1.7), 7-point Likert scale (1=strongly disagree, 7=strongly agree). In addition, German surgeons had stronger sense of time-consuming consultations that Omani surgeons, t (106) = −4.03, P <.001, N=108 surgeons (Masters et al., 2020). - 60.9% of physicians agreed or strongly agreed that IIP took up more of their time, 5-point Likert scale (1=strongly disagree, 5=strongly agree). 52.9% of them indicated that they do not have enough time to discuss internet health information with the patient, N=493 (Kim & Kim, 2009). - Physicians somewhat agreed that IIP leads to more time-consuming consultations (M=5.01, SD is not available), 7-point Likert scale (1=strongly disagree, 7=strongly agree), N=287 (Moick & Terlutter, 2012). - Physicians indicated that the duration of a medical consultation sometimes (39% of physicians) or often (39% of physicians) increases due to patients’ Internet searches, 5-point Likert scale (1=almost never, 5=almost always), 236≤N≤238 (Van Uden-Kraan et al., 2010). - Physicians indicated that discussions with IIP are sometimes longer (M=3.33, SD=1.01) and they feel that IIP are more demanding (M=3.77, SD=1.03) than normal patients, 5-point Likert scale (1=never, 5=always), N=48 (Ohana & Barnoy, 2019). - 98% of oncologists stated that the time spent discussing Internet health information had increased in the past 5 years (from 1996 to 2001), N=266 (Helft et al., 2003). - 64% of physicians believed that internet use leads longer consultations, N=375 (Potts & Wyatt, 2002). |
|  | Patients’ inappropriate medical requests | Physicians receive more inappropriate medical requests from IIP (e.g., examinations or referrals to specialists) (Wangler & Jansky, 2020).  *A huge problem is that many patients no longer go along with an unbiased medical examination but more or less want to have their search results and their conclusions confirmed. There are complex examinations or certain referrals to medical specialists being demanded of me. [...] If you as a doctor do not oppose that you will find yourself in a questionable role* [GP, male] (Wangler & Jansky, 2020). | - 44.1% of physicians agreed or strongly agreed that internet health information promotes unnecessary visits to physicians, and 26.6% gave neutral responses, 5-point Likert scale (1=strongly disagree, 5=strongly agree). Besides, 72.9% of physicians felt that IIP’s request was not appropriate, and only 16.8% reported that the patient’s request was appropriate to their health N=493 (Kim & Kim, 2009). - 59% of the physicians felt that patients raised more unreasonable requests, 5-point Likert scale (1=almost never, 5=almost always), 236≤N≤238 (Van Uden-Kraan et al., 2010). - 68% of physicians felt that IIPs’ request was not appropriate for their health, N=406 physicians who encountered an IIP in their last medical visit (Murray et al., 2003). |

**Table S2. Physicians’ communicative strategies for IIPs and perceived difficulties and needs.**

|  | | **Description** | **Supporting survey results** |
| --- | --- | --- | --- |
| **Communicative strategies for IIP** | | | |
| *Participative approach* | | | |
|  | Appreciate internet searches | Physicians acknowledge the value of internet health information and appreciate patients’ internet searches (MacDonald et al., 2018; Shachar, 2022; Wangler & Jansky, 2020).  *You know, I cannot stop my patients from looking things upon the Internet anyway. I cannot control that. Therefore, I have to make sure that something changes in the mind of the patient, even if he/she searches further. In my opinion, this is best when you signal to the patient that you listen carefully and take his/her concerns seriously. Involving the patient’s search into the conversation is always a good idea. From there it is much easier to earn the trust that is necessary* [GP, female] (Wangler & Jansky, 2020). | - Physicians reported that they sometimes appreciate internet content and encourage IIP to keep use it (M= 2.67, SD= 0.87), 5-point Likert scale (1=never, 5=very often), N=96 (Fujioka & Stewart, 2013). |
|  | Examine information | Physicians discuss and exam the internet health information brought by the patients (Ahluwalia et al., 2010; Fredriksen et al., 2018; Shachar, 2022).  *Often people find information you don’t have. Why? We’re doctors, we’re constantly being updated, [but] we’re not up to date on everything. We don’t know everything about everything. … I actually like it when someone comes and tells me something I know nothing about, and I leave his room, and I go straight to the computer … and start reading about what they said. And if it’s relevant, I can sometimes find myself incorporating [it] into my work. Definitely. … They come, they tell you something, I’ve never heard about it, I’m willing to check. And then after you check, you become a slightly better doctor. Because you know more.* [Cardiologist] (Shachar, 2022). | - 93 % of physicians agreed or totally agreed that they usually promise to verify the information from the internet and get back to the patient with an answer. 4-point Likert scale (1=totally disagree, 4=totally agree), N=116 (Giveon et al., 2009). |
|  | Acknowledge limited expertise | Physicians acknowledge their limited knowledge on certain internet health information that brought by the patients (Ahluwalia et al., 2010; Ahmad et al., 2006; MacDonald et al., 2018; Shen et al., 2015).  *Well, I actually can’t comment very much on, you know, those issues because it’s more, I think, surgical or plastic surgery more than our area* [Oncologist] (Shen et al., 2015). | Not available |
|  | Explain diagnosis/treatment | Physicians give clear clarification to patients’ diagnosis or treatment (Caiata-Zufferey & Schulz, 2012; Shachar, 2022; Shen et al., 2015).  e.g., An oncologist gives a clear explanation about hormone treatment to a patient who found internet health information about the side effects of the hormone and demonstrated concern regarding her own treatment:  *So yes it’s not pleasant to have hot flashes, it’s not pleasant to have vaginal dryness, and dry skin and all of those things. And many patients will say, ‘You’ve made an old woman out of me.’ But I think, keeping in mind, your age…the reason we started it is because of your young age – under 30. Having an estrogen receptor positive tumor means that it’s hormone driven. And when you’re pre-menopausal, you’ve got a lot of estrogen around. And it may enhance the tamoxifen. I mean there’s European data saying that ovarian suppression and tamoxifen is better than tamoxifen alone* [Oncologist] (Shen et al., 2015). | Not available |
|  | Understand patients’ emotional needs | Physicians try to understand patients’ motivations of Internet searches, their underlying concerns and preferences in treatment (Caiata-Zufferey & Schulz, 2012; Shachar, 2022).  *I simply say: “Explain to me your problem.” I try to focus on their problem. Then we examine, we evaluate, we reject hypotheses and we make the diagnosis. ... We do not discuss the validity of the Internet. In the end, retrieved information is marginal. I try to give patients a hand, I try to better understand them, we make the diagnosis together, and we decide the therapy together.* [Urologist, male, 44 years old] (Caiata-Zufferey & Schulz, 2012). | Not available |
|  | Build ongoing relations | Build an ongoing relationship with the IIP that based familiarity and trust (Ahluwalia et al., 2010; Shachar, 2022).  e.g., a cardiologist describe the experience with a patient he had been following for ten years:  *And two months ago … he had all sorts of ailments [and] went and did some tests. All the tests [were] negative, myocardial imaging … echocardiogram, and so on, and I said, “No. I think you have a problem. We should do a cardiac catheterization.” So, even though every single test was normal … I still thought he had an occlusion … and we did a catheterization, and he did have an occlusion. … So, this man, because he trusted me, because he knew me, …knew why I was insisting. … Listen, I know these people. With a lot of my patients, I know when the problem starts. The real problem. And then, yes, many times I take shortcuts. I say, “You know what, I already know you, and I know … and I’m willing to move past the imaging stage or the stress echo and do a catheterization immedi- ately.” Because I already know him*. [Cardiologist, male] (Shachar, 2022) | Not available |
|  | Instruct appropriate internet use | Physicians instruct patients to find appropriate internet health information sources and recommend reliable internet health information sites (Ahluwalia et al., 2010; Ahmad et al., 2006; Caiata-Zufferey & Schulz, 2012; Fredriksen et al., 2018; Győrffy et al., 2020; MacDonald et al., 2018; Schick et al., 2023; Shachar, 2022).  e.g., An oncologist gave explicit advice on how to use internet to find cancer information:  *So when you’re looking at things on the internet, you have to really know the sites and know like what sites are at the top of the list just depends on the number of people who hit that particular link and so forth.* [Oncologist] (Shachar, 2022).  *Yeah for me, for instance, the use of sites, I know patients when they come to you and you have to provide information they usually get shocked first to get a diagnosis and second to start treatment. And so I give them readings. I print some information for them and tell them if they have more questions to go to these sites and then you come back with me and we can discuss it if you want.* [Physician clinician-scientist, male, 29 years in practice] (MacDonald et al., 2018). | - Physicians reported that they sometimes recommend IIP to one or two internet sites (M=2.67, SD=1.21), 5-point Likert scale (1=never, 5=very often), N=96 (Fujioka & Stewart, 2013). |
| *Defensive approach* | | | |
|  | Decline to discuss | Physicians refuse to discuss internet health information by showing their resistance or referring to other specialists (Ahmad et al., 2006; Caiata-Zufferey & Schulz, 2012; Wangler & Jansky, 2020).  *Patients who begin to talk about the Internet, I don’t make a long speech. I mean, if I see that they think they know more than me, I let them understand that they can go and be treated by Mister Web.* [Surgeon, male, 60 years old] (Caiata-Zufferey & Schulz, 2012)  *If they come in and it’s too much and it’s too specialized…I let them slug it out with the specialist. They’re paid very special money to do this kind of work* [A focus group of family physician] (Ahmad et al., 2006). | Not available |
|  | Discredit/devalue internet health information | Physicians discredit the Internet as an unreliable information source and devalue the internet health information brought by the patients (Caiata-Zufferey & Schulz, 2012; Fredriksen et al., 2018; Wangler & Jansky, 2020).  *When patients tell me, “yes, but on the Internet” ..., I always cut short: “On the Web you find everything and its opposite, so forget it all and listen to what I’m saying, which is the standard.”* [Gynecologist, male, 63] (Caiata-Zufferey & Schulz, 2012).  *I do not encourage them to go online. I believe that I can give them the information they need myself. I believe that this is my strength: via individual treatment I can give them exact information about their individual complaints and symptoms* [Physiotherapist] (Fredriksen et al., 2018). | - Physicians reported that they sometimes tell patients that some online information sites are incredible (M=3.02, SD=1.16), some online information is not good (M=3.09, SD=1.07) and the risk of internet health information (M=3.22, SD=1.05). 5-point Likert scale (1=never, 5=very often), N=96 (Fujioka & Stewart, 2013). |
| **Physicians’ difficulties and needs** | | | |
|  | Extra responsibility | Extra responsibility as an information interpreter or examiner was added to physicians’ traditional professional practices (Ahmad et al., 2006; Fredriksen et al., 2018; Győrffy et al., 2020; MacDonald et al., 2018; Schick et al., 2023; Sommerhalder et al., 2009).  *When the Internet came up, many thought that patients could handle it independently, and that there was no need for us doctors. But, the opposite was the case: Our advice is getting more and more necessary in relation to this vast amount of information.* [Male physician, medical degree in 1975] (Sommerhalder et al., 2009).  *Patients are doing a lot of their own research now. Often they’ll come in and say, “What do you think of this that I read about? What do you think of that?”…* [ Family physician, male, 2 years in practices] (MacDonald et al., 2018). | - 90% of physicians perceived that most patients who had brought the internet health information because they wanted the physician's opinion on it, N=430 physicians who encountered an IIP in their last medical visit (Murray et al., 2003). - 43.0 % of physicians felt that IIP want to have physicians' opinions on internet health information they found, N=493 (Kim & Kim, 2009). |
|  | Trainings to manage IIP | Physicians seek to a training that helps them to deal with extreme cases (e.g., cyberchondria patients) and form good relations with IIP (Győrffy et al., 2020; Wangler & Jansky, 2020).  *It is completely unrealistic that we address all patients who have excessive Internet consumption. No, what we need is a tool or early-warning system which allows us to filter out exactly those patients who develop truly dysfunctional, pathological Internet use and are at risk of serious mental illness. It must be considered how we can achieve this. Everyone has a lot of catching up to do here. It will only succeed in a combination of training, education, and probably more mental health services [...] and by better networking with them.* [Family physician, male] (Wangler & Jansky, 2020). | Not available |
|  | Stay updated with technology and identify reliable internet sites | Physicians seek to be updated with technology and trustworthy Internet sites that can be referred to their patients (Ahmad et al., 2006; Schick et al., 2023; Van Uden-Kraan et al., 2010; Wangler & Jansky, 2020).  *It is imperative that doctors are trained in Internet usage. I rarely know which website to recommend to patients.* [Not available] (Van Uden-Kraan et al., 2010). | - 53% of physicians found it very/quite difficult to stay up to date with reliable health- related Internet sites for patients, 5-point Likert scale (1=very difficult never, 5=very easy), 236≤N≤238 (Van Uden-Kraan et al., 2010). - 58% of physicians agreed or totally agreed that training regarding usage of the internet is needed, and 83% of them indicated that the employer should provide internet training, if it would be available, during working hours. 4-point Likert scale (1=totally disagree, 4=totally agree), N=116 (Giveon et al., 2009). |
