# Supplementary material for: Physician Perspectives on Internet-Informed Patients: Systematic Review
Source: J Med Internet Res. 2024 Jun 6;26:e47620. doi: 10.2196/47620 (PMC11190621; doi:10.2196/47620)
Supplement: Multimedia Appendix 3 [file jmir_v26i1e47620_app3.docx]

**Appendix 3. Quality assessment**

**Table S1. CASP checklist for qualitative research**

|  | Wangler & Jansky, 2020 | Ahmad et al., 2006 | Caiata-Zufferey & Schulz, 2012 | Ahluwalia et al., 2010 | Győrffy et al., 2020 | Shachar, 2022 | Sommerhalder et al., 2009 | Shen et al., 2015 | Fredriksen et al., 2018 | MacDonald et al., 2018 | Schick et al., 2023 |
| --- | --- | --- | --- | --- | --- | --- | --- | --- | --- | --- | --- |
| Was there a clear statement of the aims of the research? | Yes | Yes | Yes | Yes | Yes | Yes | Yes | Yes | Yes | Yes | Yes |
| Is a qualitative methodology appropriate? | Yes | Yes | Yes | Yes | Yes | Yes | Yes | Yes | Yes | Yes | Yes |
| Was the research design appropriate to address the aims of the research? | Yes | Yes | Yes | Yes | Yes | Yes | Yes | Yes | Yes | Yes | Yes |
| Was the recruitment strategy appropriate to the aims of the research? | Yes | Yes | Yes | Yes | Yes | Yes | Yes | Yes | Yes | Yes | Yes |
| Was the data collected in a way that addressed the research issue? | Yes | Yes | Yes | Yes | Yes | Yes | Yes | Yes | Yes | Yes | Yes |
| Has the relationship between researcher and participants been adequately considered? | No | No | No | No  (Authors stated their own role, but no consideration of potential bias.) | No | Yes | No | No | No | No | No |
| Have ethical issues been taken into consideration? | Yes | Yes | Yes | Yes | Yes | Yes | Yes | Yes | Yes | Yes | Yes |
| Was the data analysis sufficiently rigorous? | Yes | Yes | Yes | Yes | Yes | Yes | Yes | Yes | No  (Description of the data analysis is relatively superficial.) | Yes | Yes |
| Is there a clear statement of findings? | Yes | Yes | Yes | Yes | Yes | Yes | Yes | Yes | Yes | Yes | Yes |
| How valuable is the research? | Valuable | Valuable | Valuable | Valuable | Valuable | Valuable | Valuable | Valuable | Valuable | Valuable | Valuable |

**Table S2. Risk of bias for quantitative research**

|  | Jeongeun & Sukwha, 2009 | Masters et al., 2020 | Van Uden-Kraan et al., 2010 | Fujioka & Stewart, 2013 | Murray et al., 2003 | Giveon et al., 2009 | Moick & Terlutter, 2012 | Potts & Wyatt, 2002 | Ohana & Barnoy, 2019 | da Mota et al., 2018 | Helft et al., 2003 |
| --- | --- | --- | --- | --- | --- | --- | --- | --- | --- | --- | --- |
| Was the study’s target population a close Representation of the national population in relation to relevant variables, e.g. age, sex, occupation? | No | No | No | No | No | No | No | No | No | No | No |
| Was the sampling frame a true or close representation of the target population? | No | No | Yes | No | No | Yes | No | Yes | No | No | No |
| Was some form of random selection used to select the sample, OR, was a census undertaken? | No | Yes | Yes | No | Yes | Yes | Yes | No | No | Yes | No |
| Was the likelihood of non-response bias minimal? | No | No | No | No | No | No | No | Yes | No | No | No |
| Were data collected directly from the subjects (as opposed to a proxy)? | Yes | Yes | Yes | Yes | Yes | Yes | Yes | Yes | Yes | Yes | Yes |
| Was an acceptable case definition used in the study? | Yes | Yes | Yes | Yes | Yes | Yes | Yes | Yes | Yes | Yes | Yes |
| Was the study instrument that measured the parameter of interest (e.g. prevalence of low back pain) shown to have reliability and validity (if necessary)? | Yes | Yes | No | Yes | No | Yes | No | No | Yes | No | No |
| Was the same mode of data collection used for all subjects? | Yes | Yes | Yes | Yes | Yes | No | Yes | Yes | Yes | Yes | Yes |
| Was the length of the shortest prevalence period for the parameter of interest appropriate? | Yes | Yes | Yes | Yes | Yes | No | No | Yes | No | No | Yes |
| Were the numerator(s) and denominator(s) for the parameter of interest appropriate? | Yes | Yes | Yes | Yes | Yes | Yes | Yes | Yes | Yes | Yes | Yes |
| Risk of bias | Low risk | Low risk | Low risk | Low risk | Low risk | Low risk | Moderate risk | Low risk | Moderate risk | Moderate risk | Moderate risk |
